# From Delay to Delivery: Improving Discharge Summary Compliance in a Child and Adolescent Mental Health Inpatient Unit

**DOI:** 10.1192/bjo.2026.11719

**Published:** 2026-06-30

**Authors:** Jasmine Fox, Matthew Chan, Edward Watson

**Affiliations:** 1 CWP Trust, Chester, United Kingdom; 2 Merseycare, Liverpool, United Kingdom

## Abstract

**Aims:**

Discharge summaries are essential for safe communication with patients, carers, community teams and general practitioners, ensuring continuity of care following psychiatric inpatient admission. Delays increase the risk of medication errors, incomplete risk assessments, safeguarding failures and missed follow-up. This project aimed to improve the timeliness of discharge summaries from an inpatient Child and Adolescent Mental Health (CAMHS) unit by achieving a 50% relative increase in the proportion of letters meeting both national (within 1 day) and local (within 7 days) standards on re-audit over a 6-month period.

**Methods:**

Baseline audit data showed compliance of 26.23% with the national 1-day standard and 54.1% with the local 7-day standard. Barriers to timely completion were explored through analysis of the previous 6 months data on delayed discharges with key themes to delay identified. Consistently multidisciplinary team (MDT) communication was found to be the primary issue, including resident doctors not being made aware of prospective discharge dates, and consultants not being informed when letters were uploaded for checking. As such, interventions included:

- Teaching discharge standards and processes at Trust induction for rotating resident doctors
- Identifying potential discharges during weekly MDT case planning to allow advance preparation of letters
- Ensuring resident doctor attendance at discharge meetings to facilitate same-day completion
- Supporting secretarial staff to send reminder emails to consultants when letter checking was delayed

**Results:**

Following implementation of these interventions, the 6-month re-audit evidenced that compliance was improved across both standards. National standard compliance (within 1 day) increased to 46.43%, an absolute improvement of 20.2% and a relative increase of 77.1%. Local standard compliance (within 1 week) increased to 89.29%, an absolute improvement of 35.19% and a relative increase of 65.0%. The project aim was met for both standards.

**Conclusion:**

Delayed discharge summaries compromise the safety of patients and others, through miscommunication, medication errors and failures in risk management. Despite clear national and local standards, compliance was nearly 75% and 50% respectively below expected standards. Following analysis of the previous 6 months of data on delayed discharges, MDT communication was identified as the key barrier to compliance at this CAMHS inpatient unit. This project demonstrates that simple, low-cost interventions focused on anticipation, preparation and communication can significantly improve discharge summary timeliness. Ongoing work includes six-monthly re-audit, continued teaching at Trust induction, and embedding discharge discussion within structured case-planning templates to sustain improvements.

